# Diverticulitis patient care during the Covid-19 pandemic in Germany—a retrospective nationwide population-based cohort study

**DOI:** 10.1007/s00423-023-03184-w

**Published:** 2023-11-25

**Authors:** Konstantin L. Uttinger, Maximilian Brunotte, Johannes Diers, Johan Friso Lock, Boris Jansen-Winkeln, Daniel Seehofer, Christoph-Thomas Germer, Armin Wiegering

**Affiliations:** 1 Department of General, Visceral, Transplant, Vascular and Pediatric Surgery at Würzburg University Hospital, Würzburg, Germany; 2 Department of Visceral, Transplant, Thoracic and Vascular Surgery Leipzig University Medical Center, Leipzig, Germany; 3 Department of General, Visceral and Oncological Surgery, St. Georg Hospital Leipzig, Leipzig, Germany; 4 Comprehensive Cancer Center Mainfranken, University of Würzburg Medical Center, Würzburg, Germany; 5 Department of Biochemistry and Molecular Biology, University of Würzburg, Würzburg, Germany

**Keywords:** Diverticulitis, Covid-19, Pandemic, Emergency surgery, Cohort study, In-hospital mortality

## Abstract

**Purpose:**

Coronavirus disease 2019 (COVID-19) impacted health care systems around the world. Despite a decrease in emergency admissions, an increased number of complicated forms of diverticulitis was reported. It was the aim of this study to analyze the pandemic impact on diverticulitis management in Germany.

**Methods:**

This is a retrospective population-wide analysis of hospital billing data (2012–2021) of diverticulitis in Germany. Patients were identified based on diagnosis (ICD10) and procedural codes to stratify by conservative and operative management. Primary outcome of interest was admission rates, secondary outcomes were rates of surgical vs conservative treatment and fraction of complicated clinical courses during the pandemic.

**Results:**

Of a total of 991,579 cases, 66,424 (6.7%) were admitted during pandemic lockdowns. Conservative treatment was the most common overall (66.9%) and higher during lockdowns (70.7%). Overall admissions and population adjusted rates of surgically treated patients decreased, the latter by 12.7% and 11.3%, corrected to estimated rates, in the two lockdowns. Surgery after emergency presentation decreased by 7.1% (*p*=0.053) and 11.1% (*p*=0.002) in the two lockdowns with a higher rate of ostomy and/or revision (+5.6%, *p*=0.219, and +10.2%, *p*=0.030). In-hospital mortality was increased in lockdown periods (1.64% vs 1.49%). In detail, mortality was identical in case of conservative treatment during lockdown periods (0.5%) but was higher in surgically treated patients (4.4% vs 3.6%).

**Conclusion:**

During lockdowns, there was an overall decrease of admissions for diverticulitis, especially non-emergency admissions in Germany, and treatment was more likely to be conservative. In case of surgery, however, there was increased risk of a complicated course (ostomy, re-surgery), possibly due to patient selection.

**Supplementary Information:**

The online version contains supplementary material available at 10.1007/s00423-023-03184-w.

## Introduction

Starting in late 2019, a new type of corona virus (SARS-CoV2) spread around the world causing coronavirus disease 2019 (COVID-19) [1]. Patients’ symptoms differed considerably, ranging from asymptomatic cases to respiratory failure, especially in the elderly and in comorbid patients [2, 3]. With little delay after its first occurrence, two waves of COVID-19 hit Germany in 2020, one starting in April and a second at the beginning of the winter, starting in October. As a reaction to these waves, national lockdowns were imposed by regional and national governments, the first of which lasted from April to June 2020 and the second from October 2020 to June 2021. Non-urgent elective medical procedures (including surgeries) were postponed to ensure treatment capacities of COVID-19 patients, and to reduce patient load in intensive care units during lockdown and COVID-19 periods [4, 5]. In addition, during lockdowns, patients avoided medical consultations to a high extent [6–8]. Among others, this led to a decrease in cancer diagnoses and therapies during the first COVID-19-related lockdowns in Germany and other countries [9–13]; for example, there was a decrease in rectum cancer resections of 21% during the first lockdown period in Germany compared to previous years, with a decrease of up to 32.1% in the elderly (older than 75 years) [13].

Globally, after a few weeks after the pandemic arose, fewer overall emergency visits were detected [14], while CT scan usage was increased in Emergency Department visits for acute abdominal pain [15], like for suspected cases of acute diverticulitis. If patients did present with acute diverticulitis, case severity tended to be higher, as it was found in some monocentric settings (e.g., 11.7% of 120 patients with an abscess diagnosis vs 4.4% of 339 in pre-pandemic times [16], also in [17, 18]), with higher all-cause mortality in some reports [19]. Remaining controversy was diminished in a scoping review, in which an increased number of complicated courses of diverticulitis was found during the SARS-CoV-2 pandemic [20]. In case of elective surgery postponement in patients with known previous diverticulitis, no apparent differences in outcomes were observed when compared to a non-pandemic cohort. One third of patients with complicated diverticulitis safely postponed their surgery compared to 83% of patients with uncomplicated diverticulitis, highlighting the need for triage [21]. However, in case first time acute diverticulitis was diagnosed within 30 days of Covid-19 disease, high complication rates, mostly perforation, were observed [22, 23]. To this date, there is no analysis of the extent to which management of diverticulitis, including surgery in emergency/elective settings, was affected by the pandemic circumstances in Germany.

The aim of this study was to investigate the impact of the COVID-19 pandemic, especially lockdown periods, on diverticulitis management in Germany, comparing population-adjusted rates (primary endpoints encompassing overall admissions, emergency admissions, conservative treatment, surgical treatment, secondary endpoints necessity of complex surgery and in-hospital course) during or in between lockdown periods to previous years.

## Methods

In this retrospective nationwide cohort-study of all patients admitted for diverticulitis (ICD K57, diverticulosis disease; including acute diverticulitis as subgroup, ICD-10-GM) in Germany, anonymized DRG (Diagnosis related groups) billing data provided by the “Statistische Bundesamt” (Federal Statistical Office in Germany) were used to identify patient records. No ethical approval was needed with regards to secondary data analysis of anonymized data in accordance with German national legislation. Data was handled in accordance with the data safety protocols imposed by the Federal Statistical Office in Germany. In Germany, all billing data of all private and public hospitals are reported centrally, providing completeness of all admissions for diverticulitis between 2012 and 2012 in Germany. Billing data represent highly scrutinized secondary data; protocols and in-detail descriptions of this implemented approach have previously been published [24, 25].

**Fig. 1:**
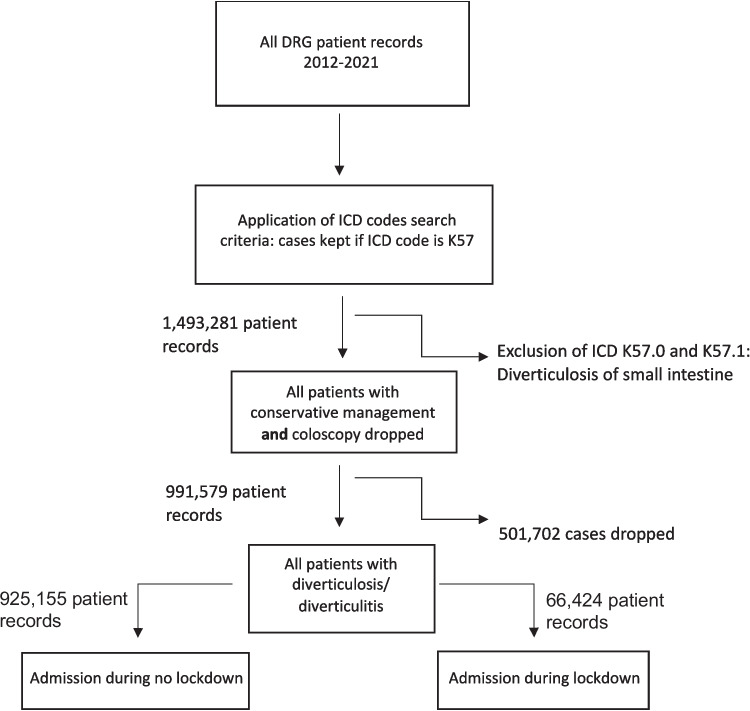
Flowchart of inclusion. Details in Supp. Table 1

Patient records in this study were identified using procedural codes (OPS; “Operationen und Prozedurenschlüssel”; Surgical and procedural coding system in Germany) codes and diagnosis codes (ICD; International Statistical Classification of Diseases and Related Health Problems) codes (Table 1 in Supplement). All patients with the main diagnosis “K57” were included, while patients with a coded diverticulitis of the small intestine (K57.0 or K57.1) were excluded. Application of these inclusion criteria identified a total of 991,579 patient records, after exclusion of patients with coloscopy only (Fig. 1).

Each patient record contained data on patient age, gender, procedure codes, all coded diagnoses, length of stay and reason for admission and discharge like emergency admission or death. In case of surgically treated patients, OPS codes were coded with corresponding time and date variables, enabling time dependent analysis of surgical performance. Procedures were considered hierarchically for each patient and the order, most importantly the first procedure (in case of multiple) was identified using date variables for each procedure code. In case of duplicates, one data set was chosen randomly. Only complete data records, except for date and time variables, were analyzed. In this analysis, we did not include complication rates or failure to rescue. This, however, would be possible on the basis of the available data and has been performed elsewhere [26–28].

There was no available information on long-term courses or readmissions, since only the index hospitalization was identified. This was due to the fact that no constant patient identifier is provided by the Federal Statistical Office.

Patients were divided in cohorts defined by the time of the Covid-19-related lockdowns of public life as well as reduction of elective surgical procedures between mid-March (22nd of March) and May (May 4th) 2020 in Germany. A first observation period with a lag of 2 weeks was defined from April 2020 to June 2020 with a pre-lockdown period from January to March 2020 (data not shown), and an interim period from July to September 2020. Also, we defined a second lockdown period beginning as a "light lockdown" from November 2^nd^, 2020 (announcement mid/end October), continuing into a lockdown until May 2021. The post-lockdown period began in June 2021 and lasted until the end of the year 2021. The same periods between 2012 and 2019 were used for reference purposes (https://www.bundesgesundheitsministerium.de/coronavirus/chronik-coronavirus.html) [13].

Incidences were calculated in rates per 100,000 people of the population (as officially published by the Federal Statistical Office per year, https://www.destatis.de/DE/Themen/Gesellschaft-Umwelt/Bevoelkerung/Bevoelkerungsvorausberechnung/_inhalt.html;jsessionid=5AFAD894070519218E9938F2ABA78B2E.live731#233982) per month to adjust for variation in the total number of people with health insurance. Mean values were reported with their standard deviation. Differences between reference and observation periods were compared using student’s t-test after testing for normal distribution. Where appropriate, 95%-confidence intervals (95% CI) were computed.

The primary endpoint in this study was the incidence, i.e., the population-adjusted rates, of admission rates as well as surgical rates (in case of surgical patients) in comparison to previous years. Secondary outcomes were rates of surgical vs conservative treatment, emergency presentations and fraction of complicated clinical courses.

Interrupted time-series (ITS) studies were conducted using an ordinary least-squared regression analysis as described elsewhere [29]. The segmentation was set between March/April 2020 and September/October 2020. Prior to regression analysis, we screened both visually and with a test as proposed by Cumby and Huizinga for autocorrelation [30].

The work has been reported in line with the STROCSS criteria [31]. It was registered retrospectively with a Research Registry UIN (researchregistry9395) (https://researchregistry.knack.com/research-registry#home/registrationdetails/64d7555ce50fe400270fa303/ ). No study protocol was published for this analysis.

All calculations were performed with Stata 16.1 (StataCorp LP, College Station, Texas, USA). A *p*-value of ≤ 0.05 was considered significant.

## Results

Between 2012 and 2021, a total of 991,579 records met the inclusion criteria (in-hospital admission for diverticulitis, ICD K57 except K57.0 and K57.1, i.e., small intestine diverticulitis, with conservative or surgical treatment) (flowchart in Fig. 1, Details in Supp. Table 1). Of these, 66,424 (6.7%) patients were admitted during Covid-19 related public lockdowns.

Descriptive data are available in Table 1, presented by lockdown status. 55.4% of all patients were female. 45.8% of patients were younger than 60 years of age, 33.0% were between 60 and 74 years old, while 21.2% were older than 74 years. In-hospital mortality was 1.5% (14,869 cases) and was higher in surgically treated patients (11,843, 3.6%). In 60.2% of cases, CT-diagnostic was performed; this fraction increased over years (51.5% in 2010, 66.7% in 2021, data not shown) and was higher during lockdown periods. The average length of stay was 8.9 days. During the lockdowns, 74.8% of patients were tested for SARS-CoV-2, of whom 605 (1.2%) tested positive. Mortality in case of SARS-CoV-2-positivy was 74 cases (9.1%), of whom in 18 cases, an Acute Respiratory Distress Syndrome was coded (data not shown) (Table 1).

**Table 1:** Patient characteristics overall and by different lockdowns

	No lockdown	Any lockdown	*P*-value
Number of patients (overall 991,579)	925,155 (93.3)	66,424 (6.7)	
Mortality (14,869, 1.5%)	13,779 (1.49)	1,090 (1.64)	*0.002*
Length of stay (mean ± sd) (overall 8.9 ± 9.2)	8.9 ± 9.2	7.8 ± 8.4	<0.001*
No. of females (overall 548,869, 55.4%)	512,536 (55.4)	36,333 (54.7)	*0.059*
0-59 years (454,191, 45.8%)	423,269 (45.8)	30,922 (46.6)	*0.015*
60-74 years (327,643, 33.0%)	305,373 (33.0)	22,270 (33.5)	*0.052*
>74 years (209,745, 21.2%)	196,513 (21.2)	13,232 (19.9)	*<0.001*
Conservative treatment (663,380, 66.9%)	616,447 (66.6)	46,933 (70.7)	*<0.001*
Mortality (3,026, 0.5%)	2,802 (0.5)	224 (0.5)	*0.483*
Surgical treatment (328,199 (33.1%)	308,708 (33.4)	19,491 (29.3)	*<0.001*
Mortality (11,843, 3.6%)	10,977 (3.6)	866 (4.4)	*<0.001*
Emergency admission (552,830, 55.8%)	510,936 (55.2)	41,894 (63.1)	*<0.001*
Mortality (10,060, 1.8%)	9,226 (1.8)	834 (2.0)	*0.008*

*P*-values stem from Chi square test except * with results from Student’s *t*-test and represent comparison between “No lockdown” and “Any lockdown.” First lockdown is admission between 04-06/2020 and second lockdown is admission between 10/2020 and 05/2021

Conservative treatment was most common overall (66.9%) and its fraction was increased during lockdowns (70.7%, *p*<0.001) with a respective significant decrease in surgically treated patients. The fraction of emergency admissions among all admissions was higher during lockdown periods (63.1% vs 55.2%, *p*<0.001). A shorter length of stay was observed during Covid-19-related lockdowns (7.8 days vs 8.9 days, *p*<0.001) (Table 1).

Overall mortality was increased in lockdown periods (1.64% vs 1.49%, *p*=0.002). In detail, mortality was identical in case of conservative treatment during lockdown periods (0.5%, *p*=0.483), but was higher in surgically treated patients in the lockdown cohort (4.4% vs 3.6%, *p*<0.001) (Table 1).

In-hospital course of events and time-dependent analysis in surgical cases are available in Table 2. In both lockdowns, fractions of interventional draining were higher (No lockdown 0.4%, lockdown 0.6%, *p*<0.001). All one-time procedures without ostomy decreased in lockdown periods (24.9% vs 19.9%, *p*<0.001). In mere descriptive analysis, no increase in fractional revision surgery (1.1% vs 1.0%, respectively) was noted, whereas mortality in case of revision surgery was increased in lockdown periods (14.9% vs 17.2%, *p*=0.286) (Table 2).

**Table 2:** Course of hospital admission: Overall and by different lockdowns

	No lockdown	Any Lockdown	*P* value
Number of patients (overall 991,579)	925,155 (93.3)	66,424 (6.7)	
Diagnostic			
SARS-CoV-2 testing (135,652, 13.7%)	85,942 (9.3)	49,710 (74.8)	*<0.001*
SARS-CoV-2 positive (815, 0.6%)	210 (0.02)	605 (1.2)	*<0.001*
CT diagnostic (597,102, 60.2%)	552,755 (60.0)	44,347 (66.8)	*<0.001*
Overall course $			
Conservative (663,380, 66.9%)	616,447 (66.6)	46,933 (70.7)	*<0.001*
Mortality (3,026, 0.5%)	2,802 (0.5)	224 (0.5)	*0.428*
Drain only (4,009, 0.4%)	3,585 (0.4)	424 (0.6)	*<0.001*
Mortality (111, 2.8%)	97 (2.7)	n.s.	*0.590*
Colon surgery only without ostomy (177,264, 17.9%)	167,521 (18.1)	9,743 (14.7)	*<0.001*
Mortality (1,303, 0.7%)	1,239 (0.7)	64 (0.7)	*0.540*
Other one-time procedure without ostomy (65,884, 6.6%)	62,443 (6.8)	3,441 (5.2)	*<0.001*
Mortality (547, 0.8%)	516 (0.8)	31 (0.9)	*0.873*
Any surgery including ostomy (20,685, 2.1%)	19,281 (2.1)	1,404 (2.1)	*0.337*
Mortality (1,895, 9.2%)	1,756 (9.1)	139 (9.9)	*0.307*
Any surgery with at least one revision surgery (10,443, 1.1%)	9,786 (1.1)	657 (1.0)	*0.072*
Mortality 1,573, 15.1%)	1,460 (14.9)	113 (17.2)	*0.286*
Surgical treatment on a weekend (6,169, 0.6%)	5,728 (0.62)	441 (0.66)	*0.358*
Mortality (460, 7.5%)	424 (7.40)	36 (8.2)	*0.841*
Time from admission to surgery	186,328 #	11,282 #	
< 12 h (32,301, 16.3% of 197,610#)	29,702 (15.9)	2,599 (23.0)	*<0.001*
Mortality (847, 2.6%)	791 (2.7)	56 (2.2)	*0.315*
12-24 h (29,878, 15.1% of 197,610#)	28,138 (15.1)	1,744 (15.5)	*0.405*
Mortality (323, 1.1%)	301 (1.1)	22 (1.3)	*0.749*
> 24 h (135,431, 68.5% of 197,610#)	128,492 (69.0)	6,939 (61.5)	*<0.001*
Mortality (2,083, 1.5%)	1,954 (1.5)	129 (1.9)	*0.078*
Timing of surgery	188,672 *	11,442 *	
Emergency hours (4.01 pm through 5 am) (15,331, 7.7% of 200,114*)	14,430 (7.6)	901 (7.9)	*0.285*
Mortality (955, 6.2%)	896 (6.2)	59 (6.5)	*0.239*
Regular hours (5.01 am through 4 pm) (182,675, 91.3% of 200,114*)	172,283 (91.3)	10,392 (90.8)	*0.889*
Mortality (2,309, 1.3%)	2,160 (1.3)	149 (1.4)	*0.244*

*P* from overall chi square. First lockdown is admission between 04-06/2020 and second lockdown is admission between 10/2020 and 05/2021. Percentages of positive SARS-CoV-2 testing refer to the whole of tested patients. *Surgical patients with available time coding of their surgical procedures mounted up to 200,114 cases, of which in 2,504, no exact date and time of admission were coded. This resulted in 197,610 cases of # surgical patients with available time coding of their surgical procedures. $ in 49,914 cases, there was an unclear course of events with regards to the classification above. ‘n.s.’ for not stated due to data protection legislation; at least one value was a maximum of 3

Surgical treatment on a weekend (0.62% in the non-lockdown-period vs 0.66% in lockdowns, *p*=0.358), surgery during emergency hours (4.01 pm through 5 am; 7.6% in the non-lockdown-period vs 7.9% in lockdowns, *p*=0.285) and very early (<12h after admission; 15.9% in the non-lockdown-period vs 23.0% in lockdowns, *p*<0.001) were increased in pandemic lockdowns, while this was statistically significant only for early surgery (Table 2).

**Fig. 2:**
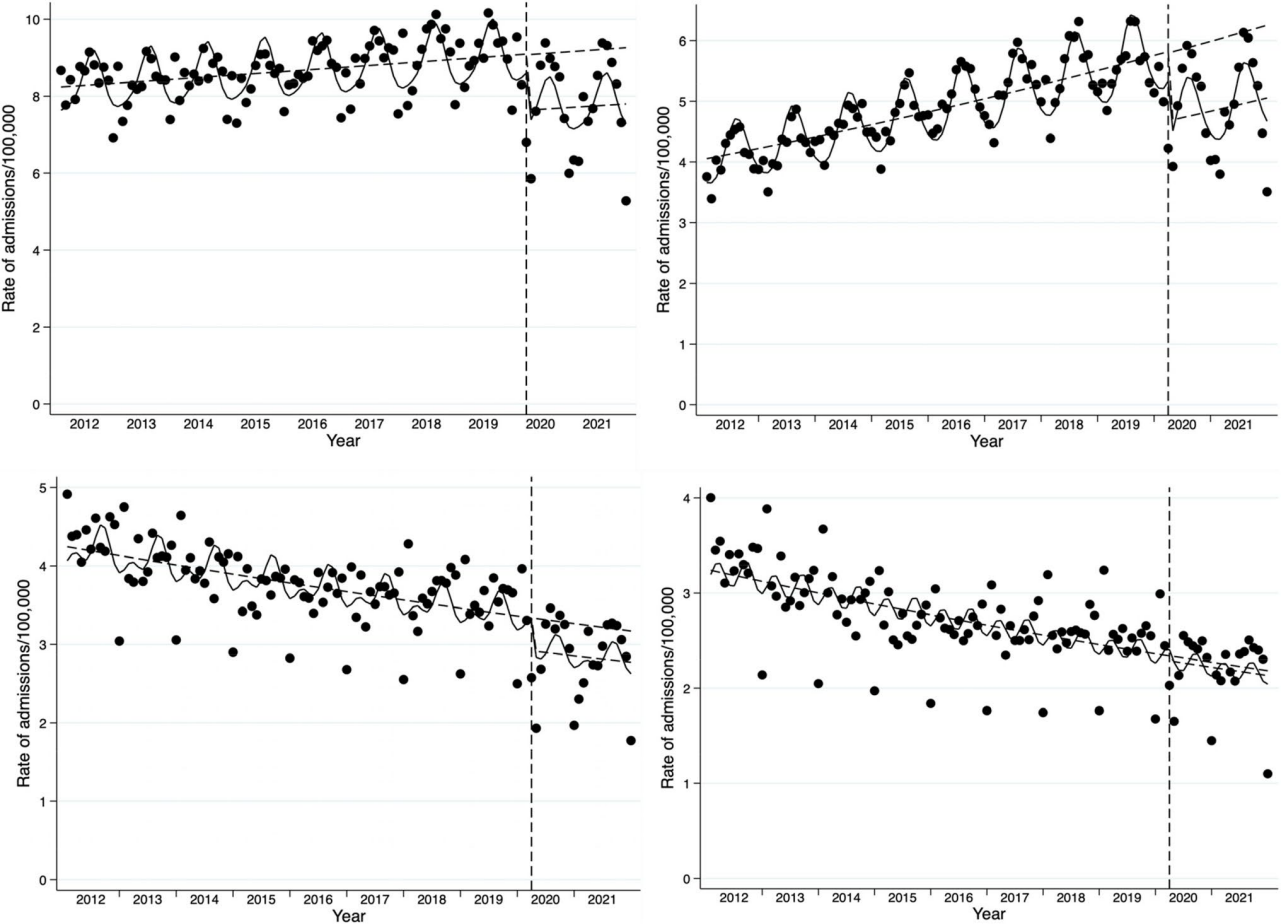
Change of standardized rates: Total (upper left, A), Emergency (upper right, B), Non-emergency admissions (lower left, C) and surgically treated patients (lower right, D) for diverticulitis. Seasonality is demonstrated using sinus-and cosinus estimates

Overall admission rates were relatively stable on monthly average over the years (min. 8.29/100,000 in 2013, max. 9.12/100,000 in 2018, min. to max. range 10.0%, Fig. 2 A) with a higher range of seasonality (min. monthly average 5.28/100,000, max. monthly average 10.2/100,000, min. to max range 93.2%). There was a constant seasonal trend to higher admission rates in summer months; this was mostly true for emergency admissions, with elective admissions peaking later in the annual cycle. Emergency admissions increased between 2012 and 2021 with a surge at the beginning of 2020 (Fig. 2 B); the opposite was the case for non-emergency admissions (4.30/100,000 in 2012, 3.62/100,000 in 2018) and surgically treated cases (3.31/100,000 in 2012, 2.58/100,000 in 2018) declining over time (Fig. 2 C and D). In light of these trends, corrected changes were calculated on the basis of the average annual change between 2012 and 2018. This average annual change was applied to the value of respective months of 2018–2019 for each lockdown to evaluate an estimate for 2020–2021. This estimation, based on an assumed linear trend, was compared to the observed rate. Admission rates and procedure rates, also stratified by emergency and non-emergency presentations, are available in Table 3 (first lockdown) and Table 4 (second lockdown). In both lockdowns, overall admissions decreased (-14.4%, *p*=0.004 in the first, corrected -24.4%, -14.6%, *p*<0.001 in the second, corrected -23.4%), while emergency admissions decreased only after adjusting for an overall increase in emergency admissions over the years (-2.8%, *p*=0.708 in the first, corrected -18.6%, -3.8%, *p*=0.386 in the second, corrected -18.1%). Overall operative procedures decreased by 23.0% (first lockdown, *p*=0.003; corrected 12.7%) and 23.6% (second lockdown, *p*<0.001, corrected 11.3%). This was more pronounced in non-emergency (elective) cases: surgical procedures decreased by 32.9% (first lockdown, *p*=0.003, corrected by estimation: 19.4%) and 31.4% (second lockdown, *p*<0.001, corrected by estimation: 19.0%). Surgery after emergency presentation decreased by 7.1% (*p*=0.053, corrected 4.3%) and 11.1% (*p*=0.002, corrected 7.2%) in the two respective lockdowns. Mortality rates remained steady in comparison to previous years during the first lockdown (-0.0%); after adjusting for the overall decline of mortality over the years of this study, an increase of 14.3% of the mortality rate during lockdown months was noted, which was also the case in the second lockdown (-6.4%, *p*=0.236, corrected +9.6%). This increase in mortality rates was higher in corrected rates of surgically treated patients (+17.6% in the first, +10.7% in the second lockdown). In surgical patients, complicated courses (i.e., need for revision surgery or ostomy) were more often during lockdowns than in reference periods of previous years. In non-emergency cases, this increase was +7.5% (first lockdown, *p*=0.144) and +1.3% (second lockdown, *p*=0.794). In emergency cases, this increase was +5.6% (first lockdown, *p*=0.219) and +10.2% (second lockdown, *p*=0.030). In population adjusted rates, however, ostomy in the course of the admission (either in the first or after revision surgery) was lower during lockdowns (Tables 3 and 4, and Supp. Table 2-4 for interim and post-lockdown periods). To confirm level and trend changes of diverticulitis patient care imposed by the Covid-19-related lockdowns in Germany, we conducted a two-step interrupted time series analysis. In a next step, we stratified this analysis by conservative and surgical admissions. All results are available in Table 5. With regards to all admissions, there was level change of -2.2 (initial intercept 8.2) for the first lockdown (*p*<0.001) and a level change of -2.8 (*p*<0.001) in the second lockdown. Level changes of smaller extents were also significant in the subgroups of conservative and surgical admissions.

**Table 3:** Admissions and procedures

	Reference to lockdown 1: Years 2012 – 2019	Lockdown 1: April 2020 through June 2020	Change	*P*
Average no of patients / month per 100,000 people	8.67 (8.46-8.89)	7.42 (3.74-11.1)	**-14.4%** **Corrected: -24.4%**	***0.004***
Mortality / month per 100,000 people	0.123 (0.12-0.13)	0.123 (0.08-0.17)	**-0.0%** **Corrected: +14.3%**	***1.0***
Ostomy / month per 100,000 people	0.64 (0.63-0.65)	0.60 (0.37-0.83)	**-6.2%** **Corrected: -7.2%**	***0.114***
Operative/interventional procedure / month per 100,000 people	2.74 (2.61-2.86)	2.11 (0.99-3.23)	**-23.0%** **Corrected: -12.7%**	***0.003***
Mortality in case of Operative/interventional procedure / month per 100,000 people	0.098 (0.09-0.10)	0.101 (0.06-0.14)	**+3.1%** **Corrected: +17.6%**	***0.733***
Emergency admissions / month per 100,000 people	4.94 (4.68-5.21)	4.80 (2.77-6.83)	**-2.8%** **Corrected: -18.6%**	***0.708***
Operative/interventional procedure in case of emergency admissions / month per 100,000 people	1.12 (1.09-1.15)	1.04 (0.80-1.28)	**-7.1%** **Corrected: -4.3%**	***0.053***
Ostomy after emergency admission / month per 100,000 people	0.384 (0.37-0.40)	0.403 (0.29-0.52)	**+4.9%** **Corrected: -5.8%**	***0.346***
Revision surgery or ostomy after emergency admission * (%)	12.4% (11.6%-13.2%)	13.1% (7.4%-22.4%)	**+5.6%**	***0.219***
Non-emergency admissions / month per 100,000 people	3.73 (3.59-3.86)	2.62 (0.97-4.28)	**-29.8%** **Corrected: -21.6%**	***<0.001***
Operative/interventional procedure in case of non-emergency admissions / month per 100,000 people	1.61 (1.51-1.72)	1.08 (0.19-1.96)	**-32.9%** **Corrected: -19.4%**	***0.003***
Ostomy after non-emergency admission / month per 100,000 people	0.253 (0.24-0.27)	0.196 (0.08-0.32)	**-22.5%** **Corrected: -11.3%**	***0.006***
Revision surgery or ostomy after non-emergency admission * (%)	8.0% (7.1%-9.0%)	8.6% (1.3%-82.5%)	**+7,5%**	***0.144***

Numbers and rates with change during periods of interest (lockdown 1). "People" represent total number of people in Germany in the respective year. p values stem from student's t-test and refer to observed rates, not corrected rates. Numbers in () represent 95% Confidence Interval. Reference always refers to the same months in the respective years. * Complicated cases were defined as any procedure including ostomy or the need for revision surgery. Absolute numbers in Supp. Table 2. Corrected values were calculated on the basis of the average annual change between 2012 and 2019. This average annual change was applied to the value of respective months of 2019 to evaluate an estimate for 2020 in assumption of a linear trend. This estimation was compared to the observed rate resulting in a corrected change. In case of mortality and ostomy, due to lower absolute numbers, three decimals are provided to avoid rounding bias

**Table 4:** Admissions and procedures

	Reference to lockdown 2: Years 2012 – 2019	Lockdown 2: October 2020 through May 2021	Change	*P*
Average no of patients / month per 100,000 people	8.43 (8.27-8.59)	7.20 (6.45-7.95)	**-14.6%** **Corrected: -23.4%**	***<0.001***
Mortality / month per 100,000 people	0.125 (0.12-0.13)	0.117 (0.11-0.13)	**-6.4%** **Corrected: +9.6%**	***0.236***
Ostomy / month per 100,000 people	0.63 (0.61-0.64)	0.60 (0.55-0.64)	**-4.8%** **Corrected: -6.7%**	***0.096***
Operative/interventional procedure / month per 100,000 people	2.80 (2.67-2.92)	2.14 (1.87-2.40)	**-23.6%** **Corrected: -11.3%**	***<0.001***
Mortality in case of Operative/interventional procedure / month per 100,000 people	0.099 (0.09-0.10)	0.092 (0.08-0.10)	**-7.1%** **Corrected: +10.7%**	***0.261***
Emergency admissions / month per 100,000 people	4.68 (4.54-4.83)	4.50 (4.07-4.92)	**-3.8%** **Corrected: -18.1%**	***0.386***
Operative/interventional procedure in case of emergency admissions / month per 100,000 people	1.08 (1.05-1.11)	0.96 (0.87-1.04)	**-11.1%** **Corrected: -7.2%**	***0.002***
Ostomy after emergency admission / month per 100,000 people	0.372 (0.36-0.38)	0.394	**+5.9%** **Corrected: -2.9%**	***0.073***
Revision surgery or ostomy after emergency admission * (%)	12.7% (12.0%-13.3%)	14.0% (11.9%-16.5%)	**+10.2%**	***0.030***
Non-emergency admissions / month per 100,000 people	3.75 (3.61-3.88)	2.70 (2.34-3.06)	**-28.0%** **Corrected: -21.7%**	***<0.001***
Operative/interventional procedure in case of non-emergency admissions / month per 100,000 people	1.72 (1.61-1.82)	1.18 (0.98-1.38)	**-31.4%** **Corrected: -19.0%**	***<0.001***
Ostomy after non-emergency admission / month per 100,000 people	0.255 (0.24-0.27)	0.202 (0.17-0.23)	**-20.8%** **Corrected: -13.3%**	***0.002***
Revision surgery or ostomy after non-emergency admission * (%)	7.5% (6.7%-8.4%)	7.6% (5.4%-10.8%)	**+1.3%**	***0.794***

Numbers and rates with change during periods of interest (lockdown 2). "People" represent total number of people in Germany in the respective year. p values stem from student's t-test and refer to observed rates, not corrected rates. Numbers in () represent 95% Confidence Interval. Reference always refers to the same months in the respective years. Absolute numbers in Supp. Table 2. Corrected values were calculated on the basis of the average annual change between 2012 and 2018 (last reference period: October 2018-May 2019). This average annual change was applied to the value of respective months of 2018-2019 to evaluate an estimate for 2020–2021 in assumption of a linear trend. This estimation was compared to the observed rate resulting in a corrected change. In case of mortality and ostomy, due to lower absolute numbers, three decimals are provided to avoid rounding bias

**Table 5:** Interrupted time series analysis

Model	Parameters	Coefficient	95%-CI, *p*-value
Overall admissions	Baseline trend	0.01	*0.004-0.01*
	Level change 04/2020	-2.22	*-2.86 - -1.59, p<0.001*
	Trend change 04/2020	0.54	*0.32 – 0.76, p<0.001*
	Level change 10/2020	-2.81	*-3.81 - -1.80, p<0.001*
	Trend change 10/2020	-0.51	*-0.80 - -0.22, p=0.001*
Conservative admissions	Baseline trend	0.02	*0.01 – 0.02, p<0.001*
	Level change 04/2020	-1.87	*-2.32 - -1.41, p<0.001*
	Trend change 04/2020	0.40	*0.24 – 0.56, p<0.001*
	Level change 10/2020	-2.29	*-3.03 - -1.56, p<0.001*
	Trend change 10/2020	-0.37	*-0.58 - -0.16, p=0.001*
Surgical admissions	Baseline trend	-0.01	*-0.01 - -0.01, p<0.001*
	Level change 04/2020	-0.36	*-0.60 - -0.11, p=0.005*
	Trend change 04/2020	0.14	*0.08 – 0.20, p<0.001*
	Level change 10/2020	-0.51	*-0.79 - -0.24, p<0.001*
	Trend change 10/2020	-0.14	*-0.22 - -0.06, p<0.001*

Impact of pandemic lockdowns on admission rates for diverticulitis. The intercept in Overall admissions was 8.24, 95% Confidence Interval, 95% CI [8.05-8.44], it was 5.02, 95% CI [4.85–5.20] in Conservative admissions and 3.22, 95% CI [3.04–3.40] in Surgical admissions. Post-trend 04/2020 in Overall admissions was 0.55, 95% CI [0.33-0.77], *p*<0.001. Post-trend 10/2020 in Overall admissions was 0.04, 95% CI [-0.09–0.1], *p*=0.529. Post-trend 04/2020 in Conservative admissions was 0.42, 95% CI [0.26–0.58], *p*<0.001. Post-trend 10/2020 in Conservative admissions was 0.05, 95% CI [-0.04–0.14], *p*=0.295. Post-trend 04/2020 in Surgical admissions was 0.13, 95% CI [0.07–0.19], *p*<0.001. Post-trend 04/2020 in Surgical admissions was -0.01, 95% CI [-0.04–0.03], *p*<0.001

## Discussion

In this retrospective population-based cohort study and time series analysis comprising a 10-year time window and close to one million patient records, we demonstrate a reduction of admissions for diverticulitis during Covid-19 lockdowns in Germany compared to previous years. Those admitted for diverticulitis during lockdowns were more likely to be conservatively treated compared to non-lockdown circumstances. The fraction of emergency admissions was increased in lockdown periods, which was due to a reduction of non-emergency admissions, since all elective procedures were reduced as imposed by national and regional governments [5, 13]. In case surgery was performed during lockdowns, there was an increase in complicated cases and, consequently, in mortality, equating to an overall increase of mortality during lockdowns in diverticulitis patients requiring hospitalization. In population adjusted mortality rates, this increase was only found after adjusting for an overall decrease of mortality over the years of this study.

Diverticulosis and diverticular disease, in acute cases referred to as diverticulitis, ranks among the most common gastroenterological entities with increasing incidence and partial chronic courses. Different stages of diverticulosis represent a complex disease with high potential for morbidity, requiring interdisciplinary collaboration to provide optimal patient care. In Germany, an interdisciplinary guideline was introduced to clarify stage-dependent diagnostic and treatment options [32]. This highlights the complexity of this patient cohort with a heterogenous comorbidity structure.

Early in the Covid-19 pandemic, a drastic decrease in emergency department presentations was noted [33, 34]. Different aspects of this decrease have to be considered. In a multicenter cohort study, for instance, Cano-Valderrama and colleagues found an increased symptom to onset time during the Covid-19 pandemic, representing possible fear of medical facilities and a possible contact with the SARS-CoV-2 virus [35]. In particular, it was recognized that many gastroenterologic entities were affected by a remarkable decrease in caseload, while, however, more urgent surgeries in case of presentation [36] and higher complication rates [16, 37] were observed. Greater case severity was found in a detailed analysis of emergency department admissions, also in patients admitted for diverticulitis [17]. Later, increased mortality rates in diverticulitis patients were noted in a large-scale analysis [19]. A pandemic impact on emergency surgery seemed to have long-lasting consequences as represented by a cross-sectional survey of 2022, in which 57.5% of survey responders of 59 countries still indicated an observation of more severe cases of diverticulitis [38].

So far, no evidence on diverticulitis patient care during the Covid-19-pandemic in Germany exists. In this analysis, we were able to demonstrate that admissions for diverticulitis during pandemic lockdowns were reduced. Those admitted for diverticulitis were more likely to be treated conservatively, which is alignment with previously published international data. The fraction of emergency admissions was increased in lockdown periods. In case surgery was performed during lockdowns, there was an increased fraction of complicated cases and increased mortality, which aligns with previous reports on diverticulitis patient care in pandemic circumstances. Weekend surgery, surgery during emergency hours and very early surgery were increased in this analysis, which was statistically significant in case of very early surgery, which can be due to different reasons. The overall non-lockdown period of this analysis comprises a long time window with presumable administrative changes over the years. Also, it is possible that in light of decreased elective surgical volume during lockdowns, there may have been more vacancy of operative capacities in case emergency surgery was necessary, not necessarily representing more urgent cases.

Increased case severity and higher complication rates can be due to different reasons. A decrease of ostomies seems to be an overall change of handling diverticulitis, and this trend was maintained in pandemic circumstances; this seems not to be in contradiction to a possible higher case severity. Intuitively, one factor for higher case severy can be longer time from symptom onset to presentation at an emergency department. It must be considered, though it was not part of this study, that a reduction of elective surgery in patients with diverticulitis may lead to complicated courses in some postponed/cancelled patients. Also, a change in daily-life habits can be assumed during lockdown periods with an impact on patient mobility and access to sportive activities. Furthermore, lifestyle adaptations were made with an increased proportion of homeworking and sitting work-environments. In addition, there is evidence that nutrition behavior was altered in a fraction of the population [39]. Mortality due to Covid-19 itself was low based on positive testing of SARS-CoV-2 incidence in the studied cohort. 6.8% of deaths in the lockdown cohort were tested positive for SARS-CoV-2, of whom only 18 had a coded Acute respiratory distress syndrome.

The major strength of the study is the comprehensive analysis of a complete population dataset reflecting the entire German population. This was independent of insurance status. Additionally, validity of the analyzed data was very high since hospital billing data is subject to intense external auditing by insurance companies.

Limitations of this study include that data was not primarily collected for scientific but for reimbursement purposes. Therefore, and due to its retrospective nature, no definitive causal inference could be made. In addition, information on disease stage and severity was missing. Data on outcome after patient discharge was not available for this analysis. Also, there was no information on patients treated in the ambulatory sector. Patients presenting to the emergency department who are diagnosed with diverticulitis and discharged due to low severity were not included in this analysis. If patients were admitted for diverticulitis more than once in the time frame of this study, they were counted as individual records. The emergency label in this analysis may exclude acute patients if they were admitted after pre-announcement by their general practitioner, or by similar channels, introducing possible bias.

In this nationwide cohort analysis, there was an overall decrease of admissions for diverticulitis in Germany during Covid-19 related lockdowns, especially non-emergency admissions. Also, there was a tendency to a higher fraction of conservatively treated patients during these periods. In case of surgery, however, there was increased risk of a complicated course during the index hospitalization (ostomy, re-surgery). Future healthcare bottleneck circumstances must respect patients with diverticulitis as prone to complicated courses with a relevant in-house mortality.

### Supplementary information

ESM 1 (DOCX 57 kb)
