# Supplementary material for: Diverticulitis patient care during the Covid-19 pandemic in Germany—a retrospective nationwide population-based cohort study
Source: Langenbecks Arch Surg. 2023 Nov 25;408(1):447. doi: 10.1007/s00423-023-03184-w (PMC10673984; doi:10.1007/s00423-023-03184-w)
Supplement: Supplementary file 1 — (DOCX 57 kb) [file 423_2023_3184_MOESM1_ESM.docx]

# Appendix

| **ICD Diagnoses** |
| --- |
| K57 (including all subgroups); K57.0 and K57.1 were excluded |
| **OPS Codes (in order of hierarchy)** |
| 5-455, 5-456, 5-458, 5-484, 5-485, 8-146, 8-148, 5-463, 5-460, 5-461, 5-541, 5-549, 5-590 |

**Supp. Table 1**: Inclusion criteria for patient records based on ICD and OPS codes.

|  | **Reference to lockdown 1: Years 2012 – 2019** | **Lockdown 1: April 2020 through June 2020** | **Change** | ***P*** |
| --- | --- | --- | --- | --- |
| **Average no of patients** / month | 7,118.1 (6,920.3-7,315.9) | 6,172.0 (3,112.0-9,232.0) | **-13.3%** | ***0.012*** |
| **Operative/interventional procedure** admissions / month | 2,241.2 (2,148.7-2,333.7) | 1,757.7 (825.4-2,690.0) | **-21.6%** | ***0.003*** |
| **Emergency admissions** / month | 4.063.3 (3,827.4-4,299.2) | 3,989.7 (2,303.5-5,675.8) | **-1.8%** | ***0.834*** |
| **Operative/interventional procedure in case of emergency** admissions / month | 918.7 (898.3-939.1) | 862.0 (661.2-1,062.8) | **-6.2%** | ***0.085*** |
| **Non-emergency admissions** / month | 3,054.8 (2,954.8-3,154.8) | 2,182.3 (807.1-3,557.6) | **-28.6%** | ***<0.001*** |
| **Operative/interventional procedure in case of non-emergency** admissions / month | 1,322.5 (1,240.0-1,405.0) | 895.7 (161.6-1,629.7) | **-32.3%** | ***0.002*** |
|  | **Reference to lockdown 2: Years 2012 – 2019** | **Lockdown 2: October 2020 through May 2021** |  |  |
| **Average no of patients** / month | 6,917.3 (6,779.7-7,054.9) | 5,988.5 (5,366.4-6,610.6) | **-13.4%** | ***<0.001*** |
| **Operative/interventional procedure** admissions / month | 2,290.2 (2,190.2-2,390.1) | 1,777.3 (1,557.0-1,996.5) | **-22.4%** | ***<0.001*** |
| **Emergency admissions** / month | 3,846.2 (3,717.4-3,975.0) | 3,740.6 (3,387.6-4,093.7) | **-2.7%** | ***0.580*** |
| **Operative/interventional procedure in case of emergency** admissions / month | 884.6 (863.9-904.2) | 795.1 (725.0-865.2) | **-10.1%** | ***0.005*** |
| **Non-emergency admissions** / month | 3,071.1 (2,966.8-3,175.4) | 2,247.9 (1,946.0-2,549.7) | **-26.8%** | ***<0.001*** |
| **Operative/interventional procedure in case of non-emergency** admissions / month | 1,405.6 (1,323.3-1,487.9) | 982.1 (815.8-1,148.5) | **-30.1%** | ***<0.001*** |

**Supp. Table 2: Admissions and procedures:** numbers and rates with change during periods of interest (lockdown periods). p values stem from student's t-test. Numbers in () represent 95% Confidence Interval. Date per 100,000 people in Table 3 and Table 4.

|  | **Previous years (01-12/2012-2019)** | **The year 2020 (01-12/2020)** | **Change: 2012-19 to 2020** | ***P*** | **The year 2021 (01-12/2021)** | **Change: 2012-19 to 2021** | ***P*** |
| --- | --- | --- | --- | --- | --- | --- | --- |
| **Average no of patients** / month | 7,116.3 (6,995.1-7,237.4) | 6,648.8 (5,985.2-7,312.4) | **-6.6%** | ***0.022*** | 6,429.3 (5,755.0-7,103.6) | **-9.7%** | ***0.001*** |
| Average no of patients / month **per 100,000 people** | 8.67 (8.53-8.81) | 8.00 (7.20-8.79) | **-7.7%** | ***0.004*** | 7.72 (6.91-8.53) | **-11.0%** | ***<0.001*** |
| **Operative/interventional procedure** admissions / month | 2,274.8 (2,204.5-2,345.0) | 1,900.3 (1,679.8-2,120.7) | **-16.5%** | ***<0.001*** | 1,824.3 (1,627.5-2,021.1) | **-19.8%** | ***<0.001*** |
| Operative/interventional procedure / month **per 100,000 people** | 2.78 (2.69-2.87) | 2.29 (2.02-2.55) | **-17.6%** | ***<0.001*** | 2.19 (1.96-2.43) | **-21.2%** | ***<0.001*** |
| **Emergency admissions** / month | 4,011.4 (3,893.5-4,129.4) | 4,159.9 (3,793.6-4,526.3) | **+3.7%** | ***0.406*** | 4,080.7 (3,627.0-4,534.4) | **+1.7%** | ***0.706*** |
| Emergency admissions / month **per 100,000 people** | 4.88 (4.75-5.02) | 5.00 (4.56-5.44) | **+2.5%** | ***0.565*** | 4.90 (4.36-5.45) | **+0.4%** | ***0.933*** |
| **Operative/interventional procedure in case of emergency** admissions / month | 902.7 (887.1-918.2) | 842.6 (780.7-904.5) | **-6.7%** | ***0.015*** | 825.3 (734.6-916.1) | **-8.6%** | ***0.004*** |
| Operative/interventional procedure in case of emergency admissions / month **per 100,000 people** | 1.10 (1.08-1.12) | 1.01 (0.94-1.09) | **-8.2%** | ***0.005*** | 0.99 (0.88-1.10) | **-10.0%** | ***0.001*** |
| **Non-emergency admissions** / month | 3,104.8 (3,031.0-3,178.6) | 2,488.9 (2,169.0-2,808.9) | **-19.8%** | ***<0.001*** | 2,348.7 (2,109.7-2,587.6) | **-24.4%** | ***<0.001*** |
| Non-emergency admissions / month **per 100,000 people** | 3.79 (3.69-3.88) | 2.99 (2.61-3.38) | **-21.1%** | ***<0.001*** | 2.82 (2.55-3.11) | **-25.6%** | ***<0.001*** |
| **Operative/interventional procedure in case of non-emergency** admissions / month | 1,372.1 (1,312.9-1,431.3) | 1,057.7 (1,279.0-1,395.4) | **-22.9%** | ***<0.001*** | 999.0 (880.4-1,117.6) | **-27.2%** | ***<0.001*** |
| Operative/interventional procedure in case of non-emergency admissions / month **per 100,000 people** | 1.68 (1.60-1.75) | 1.27 (1.07-1.48) | **-24.4%** | ***<0.001*** | 1.20 (1.06-1.34) | **-28.6%** | ***<0.001*** |

**Supp. Table 3: Admissions and procedures:** numbers and rates with change during the whole year 2020. "People" represent total number of people in Germany in the respective year. p values stem from student's t-test. Numbers in () represent 95% Confidence Interval. No correction calculation was performed.

|  | **Interim period (reference) (07-09 2012-2019)** | **Interim period (observation) (07-09 2020)** |  |  |
| --- | --- | --- | --- | --- |
| **Average no of patients** / month | 7,571.5 (7,371.8–7,771.2) | 7,520.7 (6,876.3-8,165.0) | **-0.7%** | **0.858** |
| Average no of patients / month **per 100,000 people** | 9.23 (9.02-9.43) | 9.04 (8.27-9.82) | **-2.1%** | **0.536** |
| **Operative/interventional procedure** admissions / month | 2,246.5 (2,156.9-2,336.1) | 2,035.7 (1,957.3-2,114.0) | **-9.4%** | **0.103** |
| Operative/interventional procedure / month **per 100,000 people** | 2.74 (2.62-2.86) | 2.45 (2.35-2.54) | **-10.6%** | **0.097** |
| **Complicated** operative cases* (%) | 9.6% (8.9%-10.4%) | 9.9% (6.3%-13.8%) | **+3.1%** | **0.348** |
| **Emergency admissions** / month | 4,369.3 (4,127.8-4,610.9) | 4,740.3 (4,179.6-5,301.0) | **+8.5%** | **0.283** |
| Emergency admissions / month **per 100,000 people** | 5.32 (5.05-5.59) | 5.70 (5.03-6.37) | **+7.1%** | **0.323** |
|  | **Postlockdown period (reference) (06-12 2012-2019)** | **Postlockdown period (observation) (06-12 2021)** |  |  |
| **Average no of patients** / month | 7,254.0 (7,082.8-7,425.2) | 6,781.0 (5,670.1-7,891.9) | **-6.5%** | **0.104** |
| Average no of patients / month **per 100,000 people** | 8.84 (8.65-9.03) | 8.15 (6.81-9.48) | **-7.8%** | ***0.040*** |
| **Operative/interventional procedure** admissions / month | 2,191.4 (2,100.3-2,282.6) | 1,841.0 (1,460.7-2,221.3) | **-16.0%** | ***0.015*** |
| Operative/interventional procedure / month **per 100,000 people** | 2.67 (2.56-2.79) | 2.21 (1.75-2.67) | **-17.3%** | **0.011** |
| **Complicated** operative cases* (%) | 9.7% (9.0%-10.4%) | 9.8% (6.2%-15.3%) | **+1.0%** | **0.766** |
| **Emergency admissions** / month | 4,182.8 (4,031.4-4,334.2) | 4,353.3 (3,630.8-5,075.8) | **+3.9%** | **0.474** |
| Emergency admissions / month **per 100,000 people** | 5.09 (4.92-5.26) | 5.23 (4.36-6.10) | **+2.8%** | **0.614** |

**Supp. Table 4:** Pre-lockdown, interim and post-lockdown period. "People" represent total number of people in Germany in the respective year. p values stem from student's t-test. Reference always refers to the same months in the respective years, observation refers to the months in pandemic years (2021 and 2021).
